# Iron Promotes Intestinal Development in Neonatal Piglets

**DOI:** 10.3390/nu10060726

**Published:** 2018-06-05

**Authors:** Yutian Pu, Shuhui Li, Haitao Xiong, Xiaofeng Zhang, Yizhen Wang, Huahua Du

**Affiliations:** 1 Key Laboratory of Animal Nutrition and Feed Science (Eastern of China), Ministry of Agriculture, Key Laboratory of Animal Feed and Nutrition of Zhejiang Province, College of Animal Science, Zhejiang University, Hangzhou 310058, China; pyt0830@126.com (Y.P.); 11617013@zju.edu.cn (S.L.); xht1003@126.com (H.X.); yzwang321@zju.edu.cn (Y.W.); 2 College of Animal Science and Technology, China Agricultural University, Beijing 100193, China; 3 Institute of Animal Husbandry and Veterinary Science, Zhejiang Academy of Agricultural Sciences, Hangzhou 310021, China; hnkjmc@163.com

**Keywords:** iron, neonatal, immunity, intestine, cytokine

## Abstract

Early nutrition is key to promoting gut growth and education of the immune system. Although iron deficiency anemia has long been recognized as a serious iron disorder, the effects of iron supplementation on gut development are less clear. Therefore, using suckling piglets as the model for iron deficiency, we assessed the impacts of iron supplementation on hematological status, gut development, and immunity improvement. Piglets were parenterally supplied with iron dextran (FeDex, 60 mg Fe/kg) by intramuscular administration on the third day after birth and slaughtered at the age of two days, five days, 10 days, and 20 days. It was expected that iron supplementation with FeDex improved the iron status with higher levels of serum iron, ferritin, transferrin, and iron loading in the liver by regulating the interaction of hepcidin and ferroportin (FPN). FeDex supplementation increased villus length and crypt depth, attenuated the pathological status of the duodenum, and was beneficial to intestinal mucosa. FeDex also influenced the intestinal immune development by stimulating the cytokines’ production of the intestine and enhancing the phagocytotic capacity of monocytes. Overall, the present study suggested that iron supplementation helped promote the development of the intestine by improving its morphology, which maintains its mucosal integrity and enhances the expression of immuno-associated factors.

## 1. Introduction

Iron deficiency is considered to be the most common mammalian nutritional deficiency among infants and young children between the ages of zero and five years in developing countries as well as in affluent societies [1]. It is also considered to be the primary cause of anemia especially in the neonatal period [2]. The main reason for iron deficiency of newborns is the lack of iron from sow’s milk, which leads to their rapid growth especially the increase in red blood cells and enterocytes [3].

Iron is a crucial element involved in many central metabolic pathways such as oxygen transport, DNA synthesis, and redox reactions [4]. A lack of iron alters the efficiency of cell proliferation and regeneration. Iron deficiency during the fetal and neonatal period will lead to dysfunction of multiple organ systems in which some might not recover in the later stages despite iron rehabilitation [5]. Therefore, maintaining iron homeostasis is essential for optimal development and function of most organ systems. Experimental evidence has shown that iron is a fundamental element for normal development of the immune system [6]. It has been proven that iron modulates immune mechanisms such as cytokine activities, immune cell proliferation, monocyte/macrophage differentiation, and malnutrition of lymphocytes [7]. Therefore, iron deficiency also affects the capacity for generating an adequate immune response to infections [8]. Bactericidal activity of macrophages was attenuated by iron deficiency [9]. T-lymphocyte number, blastogenesis, and mitogenesis were decreased in iron deficiency in response to several different mitogens [6]. Neutrophils had a reduced activity for intracellular killing of pathogens in iron deficiency [10]. 

Newborn piglets are suitable models for iron deficiency because they are born with limited iron storage, which is similar to humans, and can quickly become iron deficient without exogenous iron supplementation [11]. Actually, iron deficiency anemia has long been recognized as a serious iron disorder in suckling piglets [12]. Since breast milk is not sufficient for meeting the iron requirement of suckling piglets, the use of intramuscular injection of exogenous iron to prevent further iron deficiency has been well established [3,13].

Therefore, the aim of this study was to determine the ability of iron supplementation to keep iron homeostasis, improve the intestinal morphology, and promote the intestinal immunity in neonatal piglets. We hypothesized that piglets that gained iron supplementation would have corresponding increases in iron status, which resulted in the maintenance of integral intestinal epithelia and the increased secretion of cytokines. These are the key factors of many immunologic steps. 

## 2. Materials and Methods

### 2.1. Piglets and Experimental Design

Piglets were provided by the Zhejiang Academy of Agricultural Sciences (Hangzhou, China). Animal experimental procedures were approved by the Institutional Animal Care and Use Committee of Zhejiang University. A total of 28 Duroc × Large White piglets were taken from 6 L delivered by 4 primiparous and 2 multiparous sows. They were housed in standard conditions of approximately 70% humidity and a temperature of 22 ± 2 °C. Four piglets were slaughtered on the second day of age as pretreatment control. Twenty-four piglets with an average body weight of 1.37 kg were randomly alloted to two groups (*n* = 12 per group): intramuscular injection with PBS (Control) or 60 mg Fe/kg body weight of iron dextran (FeDex) on the third day of age. Four piglets per treatment were slaughtered at the age of 5 days, 10 days, and 20 days. Blood samples were collected from the anterior vena cava before euthanasia and serum was obtained after centrifugation at 3000× *g* for 10 min at 4 °C. Piglets were sacrificed by CO_2_ inhalation and exsanguination. The samples of the liver, spleen, middle duodenum, and distal ileum were collected and frozen in liquid nitrogen and then held at −80 °C until they were analyzed. The serum samples were analyzed using chemical-based colorimetric assay kits (Abcam, Shanghai, China) to evaluate the levels of d-lactic acid and diamine oxidase (DAO).

### 2.2. Iron Measurement and Hepatic Iron Staining

Erythrocyte counts and erythrocyte parameters as well as serum iron levels were determined by using an automated SYSMEX F820 Analyzer (Sysmex, Shanghai, China). The Enzyme Linked Immunosorbent Assay (ELISA) Kits (Abcam, Shanghai, China) were performed to measure the concentrations of ferritin and transferrin. Iron accumulation in the liver was measured by staining with Prussian blue. The liver was fixed in Bouin’s solution at 20 °C for 72 h and then stored in 70% ethanol. After dehydration, the fragments were embedded in paraffin and then cut into 5 μm sections. After mounting on glass slides, sections were deparaffinized, stained with Perls’ Prussian blue, counterstained with nuclear red, and analyzed under a light microscope (Zeiss, Jena, Germany).

### 2.3. Real-Time PCR Analysis

Total RNA was extracted by using Trizol Reagent kits (Sigma, Beijing, China). RNA quantity and purity were determined using a NanoDrop 2000 spectrophotometer (Thermo Fisher Scientific, Waltham, MA, USA). Real-time PCR was conducted using iQTW5 real-time multiplexing system (Bio-Rad, Shanghai, China). M-MuLV Reverse Transcriptase (Thermo Fisher Scientific, Shanghai, China) and oligo primer transcriptase were used to reverse RNA to cDNA. The following primers were used: 18S forward 5′-CCCACGGAATCGAGAAAGAG’ and reverse 5′-TTGACGGAAGGGCACCA-3′; Hepcidin forward 5′-GAGCCACCGCTGGTTTGAC-3′ and reverse 5′-ACATCCCACAGATTGCTTTGC-3′; Interleukin (IL)-6 forward 5′-TGGCTACTGCCTTCCCTACC-3′ and reverse 5′-CAGAGATTTTGCCGAGGATG-3′; Interferon-γ (IFN-γ) forward 5′-CAAAGCCATCAGTGAACTCATCA-3′ and reverse 5′-TCTCTGGCCTTGGAACATAGTCT-3′; IL-1β forward 5′-ACAAAAGCCCGTCTTCCTG-3′ and reverse 5′-ATGTGGACCTCTGGGTATGG-3′; Transforming growth factor-β (TGF-β) forward 5′-ACGTGGZGCTAYACCAGAAATACAG-3′ and reverse 5′-ACAACTCCGGTGACATCAAAGG-3′; Porcine beta-defensin (pBD)-1 forward 5′-TGCCACAGGTGCCGATCT-3′ and reverse 5′-CTGTTAGCTGCTTAAGGAATAAAGGC-3′; pBD-2 forward 5′-CCAGAGGTCCGACCACTACA-3′ and reverse 5′-GGTCCCTTCAATCCTGTTGAA-3′. Fold changes were calculated after normalizing the change in expression of the gene of interest to the housekeeping gene 18S using the threshold cycle values. 

### 2.4. Histomorphology Analysis

Hematoxylin and eosin (H&E) staining was performed to determine the morphology of duodenum tissue. Duodenum cross-sections were placed overnight in fixative containing 10% formaldehyde. Samples were then paraffin-embedded and cut into 5 μm pieces in the longitudinal plane and mounted on glass slides. Slides were stained with H&E using standard techniques. Sections were examined under a DM3000 microscope (Leica, Wetzlar, Germany). Villous height and crypt depth were measured using Image-Pro software (Leica, Wetzlar, Germany).

### 2.5. Western Blot Analysis

The total protein was extracted from duodenum and spleen tissues and quantified using the Pierce BCA Protein Assay Kit (Thermo Fisher Scientific). Samples (50 μg) were electrophoresed through a 10% SDS-PAGE followed by electro-transferred to polyvinylidene fluoride membranes (Millipore, Billerica, MA, USA). After blocking in defatted milk powder, the membranes were incubated with an anti-swine ferroportin (FPN) antibody (1:2000 dilution, Abcam, Shanghai, China) and an anti-swine β-actin antibody (1:2000 dilution, Sigma, Beijing, China) followed by an incubation in the presence of a peroxidase-labeled secondary antibody (Pierce, Rockford, USA). Blots were visualized by using a chemiluminescence detection kit (CliNX, Shanghai, China).

### 2.6. Phagocytosis Assay 

To analyze the phagocytic activity of neutrophils and monocytes, the peripheral blood mononuclear cells (PBMC) were incubated with fluorescein isothiocyanate-(FITC-) dextran (1 mg/mL) at 37 °C for 1 h. After incubation, the cells were washed twice with PBS and the percentage of intracellular FITC-dextran was determined by the Fluorescence Activating Cell Sorter (FACS).

### 2.7. Statistical Analysis

All data are presented as means and standard deviations. Each animal was considered an experimental unit. Statistical analyses between two treatments or within the same treatment over time were performed using a two-way analysis of variance (ANOVA) with a Tukey’s *post hoc* correction. A *p*-value < 0.05 was considered statistically significant.

## 3. Results

### 3.1. Iron Supplementation Improved Iron Status of Piglets

**Figure 1:**
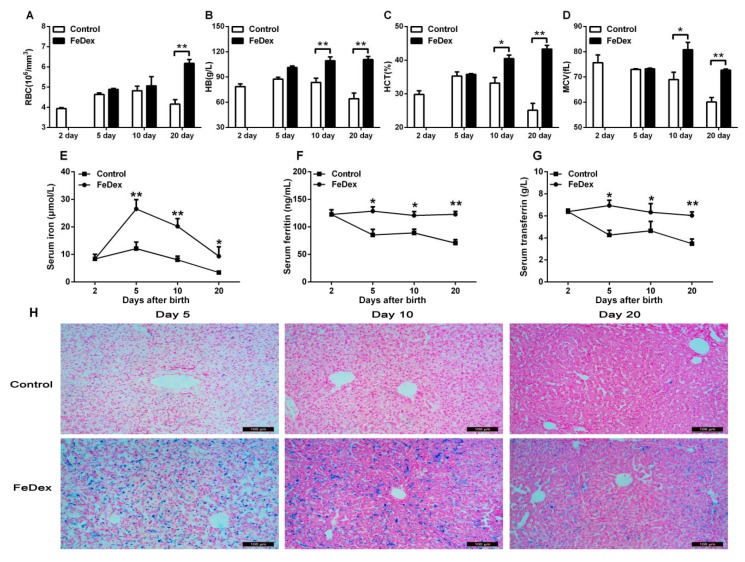
Hematological parameters and iron status in piglets. Red blood cell (RBC) count (**A**), hemoglobin (Hb) concentration (**B**), hematocrit (HCT) value (**C**), mean cell volume (MCV) (**D**), the concentration of iron (**E**), ferritin (**F**), and transferrin (**G**) in serum were determined for four piglets from each group at each time point. Non-heme iron deposits in liver (**H**) were detected by staining with Perls’ Prussian blue and the cells counterstained with nuclear red. Values are expressed as the mean ± SD. Asterisks indicate a significant difference (* *p* < 0.05; ** *p* < 0.01) in comparison with control piglets’ values at the given day after birth.

Evaluation of the hematological parameters of piglets at the age of two days clearly showed that these piglets were verging on borderline anemia (see Figure 1A–D). Without iron supplementation, the hematological status of control piglets gradually and significantly worsened. On the age of 20 days, low values for red blood cell (RBC) count, hemoglobin (HB) concentration, hematocrit (HCT) percentage, and mean cell volume (MCV) indicated the occurrence of severe iron deficiency anemia (see Figure 1E). FeDex supplementation efficiently prevented the deterioration of the hematological status of newborn piglets and contributed to the recovery of animals from the pre-anemic state.

The levels of iron, ferritin, and transferrin in serum were examined to evaluate the iron status. Compared with control piglets, FeDex-supplemented piglets had higher concentrations of iron, ferritin, and transferrin in serum (*p* < 0.05) (see Figure 1E–G). The serum iron was significantly increased (*p* < 0.01) on day five after birth and then it slowly reduced along with the growth of piglets (see Figure 1E). However, there was no difference for serum ferritin and transferrin in different ages of FeDex-supplemented piglets (see Figure 1F–G).

The liver is the main site of iron storage. Iron released from FeDex is mainly ingested by Kupffer cells in the liver [14]. Microscopic analysis of liver sections stained for non-heme iron with Perls’ Prussian blue showed that heavy iron deposited in the Kupffer cells of piglets supplemented with FeDex (see Figure 1H). The iron accumulation was observed 120 h after the injection of FeDex (Day 5) and was maintained until the end of the experiment (Day 20). Non-heme iron deposits were all increased in the liver of FeDex-supplemented piglets. Furthermore, the deposits were decreased with the growth of piglets in both groups. 

### 3.2. Relationship between Iron Status of Piglets and Hepcidin mRNA Level

**Figure 2:**
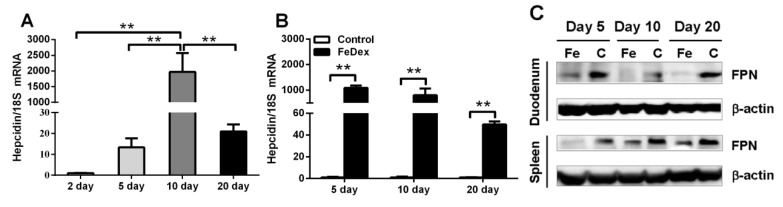
Hepcidin mRNA abundance and FPN protein levels in piglets. Hepcidin transcripts changes with the growth in FeDex-supplemented piglet livers (**A**) and comparison of two groups (**B**) by real-time RT-PCR. Each point represents the mean ± SD two amplification reactions for liver samples from four piglets. Values were normalized against the level of 18S rRNA. The mRNA level of Hepcidin in FeDex-supplemented piglets on the second day (**A**) and Hepcidin in control piglets (**B**) was assigned the value of 1. Asterisks indicate significant difference (** *p* < 0.01) in comparison with control piglets’ values at the given day after birth. FPN levels in the duodenum and spleen was assessed by the Western blot (**C**). Blots were re-probed with monoclonal anti-β-actin antibody as a loading control. The presented data are representative of Western blot analyses of duodenum and spleen obtained from four piglets at each time point.

Iron is the first biological factor shown to induce hepcidin expression and there is experimental evidence that both high iron saturation of plasma transferrin and hepatic iron loading stimulate hepcidin synthesis in liver [15]. In this study, when supplemented with FeDex, hepcidin transcription was significantly increased (*p* < 0.01) on day 10 after birth and then it slowly reduced along with the growth of piglets (see Figure 2A). The mRNA expression profile of hepcidin was similar to the changes of serum iron levels (see Figure 1E). At the same time, FeDex-supplemented piglets showed much higher (*p* < 0.01) mRNA expression of hepcidin (see Figure 2B). Hepcidin can mediate iron homeostasis by binding to the iron exporter FPN, which induces its internalization and degradation [16]. FPN levels in duodenum and spleen were measured to determine their relationship with hepcidin expression and molecular potential for iron exportation. The observed decrease of FPN expression in the duodenum and spleen of FeDex-supplemented piglets was in accordance with an opposite increase in hepcidin levels (see Figure 2C). 

### 3.3. Iron Supplementation Attenuated the Damage of Duodenal Mucosal

**Figure 3:**
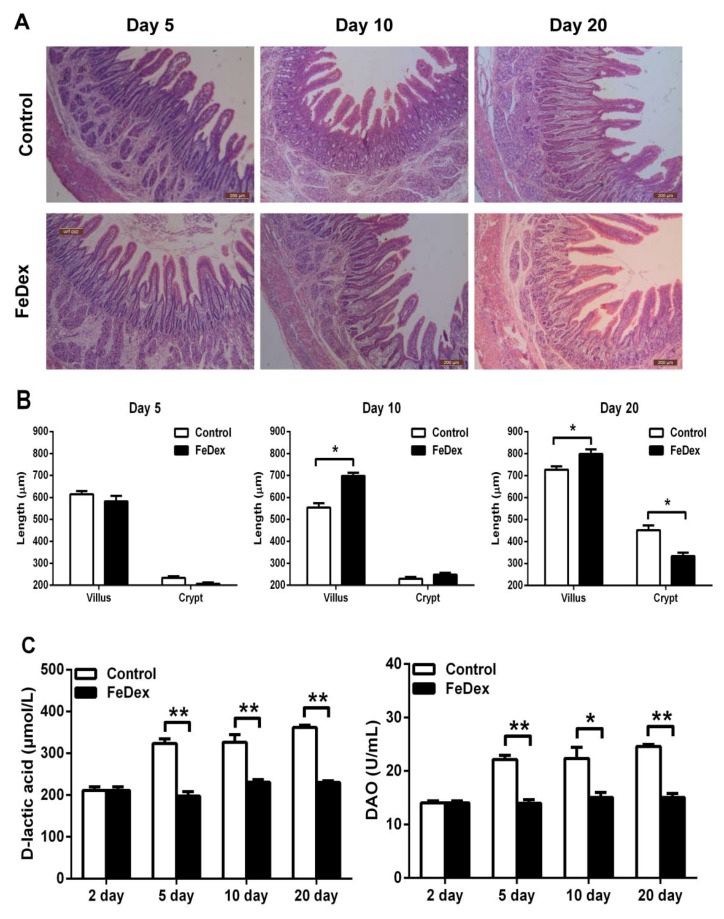
Morphological comparisons of the duodenum mucosa between control and FeDex-supplemented piglets. (**A**) Duodenum mucosa epithelium (*n* = 4) from each group were processed for morphological evaluation. Scale bar = 200 μm. (**B**) The relative villus and crypt length were measured. There are four measurements in each time point and villous height and crypt depth were measured using Image-Pro software. Asterisks indicate that the mean value of FeDex-supplemented group was significantly different from that of the other control group (*p < 0.05). (**C**) Serum levels of d-lactic acid and DAO in control and FeDex-supplemented piglets. Values are expressed as the mean ± SD for serum samples obtained from four piglets at each time point. Asterisks indicate a significant difference (* *p* < 0.05, ** *p* < 0.01) in comparison with control values at the given day after birth.

H&E staining was used to determine the effects of iron on the duodenum morphology development. With growth, the length of duodenal villus increased and the duodenal wall became thicker (see Figure 3A). On the fifth day, there was no significant difference in the relative lengths of villus and crypt between the control and FeDex-supplemented piglets (see Figure 3B). On day 10, the villus length in FeDex-supplemented piglets was significantly longer than that of control piglets (*p* < 0.05) while no significant difference was observed in crypt length (see Figure 3B). On day 20, villous atrophy and serious porosity of the submucosa layer were observed in the duodenum of control piglets. However, the growth of villus in FeDex-supplemented piglets was better with an increased ratio of villus height to crypt depth (*p* < 0.05) (see Figure 3B). The serum levels of d-lactic acid and DAO are important biochemical markers for evaluating intestinal mucosal structure and function. Iron deficiency caused significantly higher levels of d-lactic acid and DAO in serum of the control group from the fifth day to the 20th day. However, FeDex-supplemented piglets had lower levels (*p* < 0.05), which were similar to untreated piglets on the second day (see Figure 3C). The result indicated that FeDex supplementation significantly attenuated the pathological status of the duodenum and was beneficial to intestinal mucosa.

### 3.4. Iron Supplementation Stimulated the Expression of Cytokines Genes of Intestine

**Figure 4:**
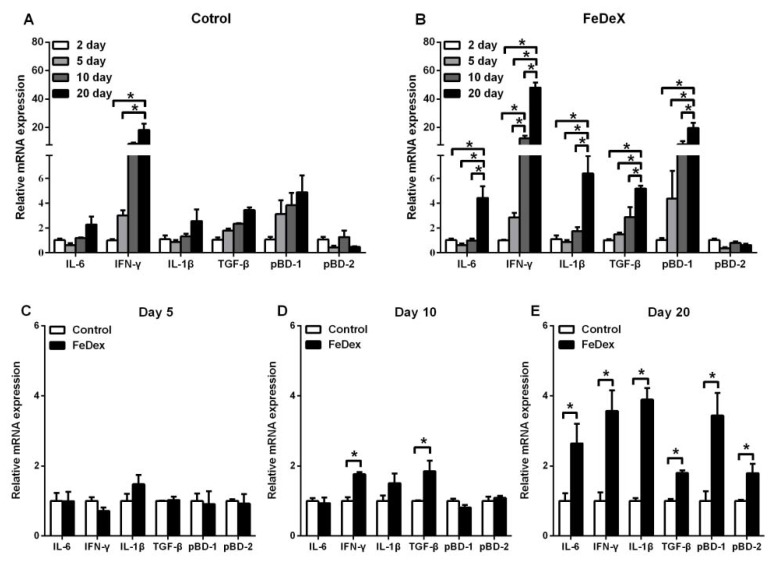
Analysis of immune-associated factors mRNA expression in the ileum of piglets. Comparison of the expression profile of inflammatory cytokines and antimicrobial peptides at different days after birth (**A**,**B**). Comparison of the expression inflammatory cytokines and antimicrobial peptides at day 5 (**C**), day 10 (**D**), and day 20 (**E**) after birth, respectively. Values were normalized against the level of 18S rRNA. The mRNA level in control piglets was assigned the value of 1. Values were normalized against the level of 18S rRNA. The level of mRNA level in FeDex-supplemented piglets two days after birth was assigned the value of 1. Each point represents the mean ± SD two amplification reactions performed on a single cDNA sample reverse-transcribed from separate RNA isolated from four piglets. Asterisks indicate significant differences (* *p* < 0.05).

The presence of cytokines likely influences development of the neonatal immune system [17]. To determine whether the supplementation of FeDex correlated with immune development of the intestine, the mRNA expressions of cytokines were measured. During the study, no diarrhea was observed in piglets, which suggested that pathological inflammation or infection was excluded. The preliminary experiment showed that ileum was the most abundant tissue to express cytokines genes (data not shown). This data showed that only IFN-γ mRNA level was elevated with age while the detected inflammatory cytokines (IL-6, IFN-γ, IL-1β, and TGF-β) and antimicrobial peptide pBD-1 in FeDex-supplemented piglets were all significantly increased (*p* < 0.05) (see Figure 4A,B). There was no difference in detected inflammatory cytokines and antimicrobial peptides between two groups on the fifth day of age (see Figure 4C). On day 10, IFN-γ and TGF-β transcripts were increased significantly (*p* < 0.05) in ileum of FeDex-supplemented piglets (see Figure 4D). On the 20th day of age, the mRNA expressions of all inflammatory cytokines (IL-6, IFN-γ, IL-1β, and TGF-β) and antimicrobial peptides (pBD-1 and pBD-2) were highly (*p* < 0.05) expressed (see Figure 4E). The increased cytokine production by mucosal and systemic immune cells from piglets supplemented with FexDex suggested a priming of the immune system.

### 3.5. Iron Supplementation Enhanced Phagocytotic Capacity of Monocytes

**Figure 5:**
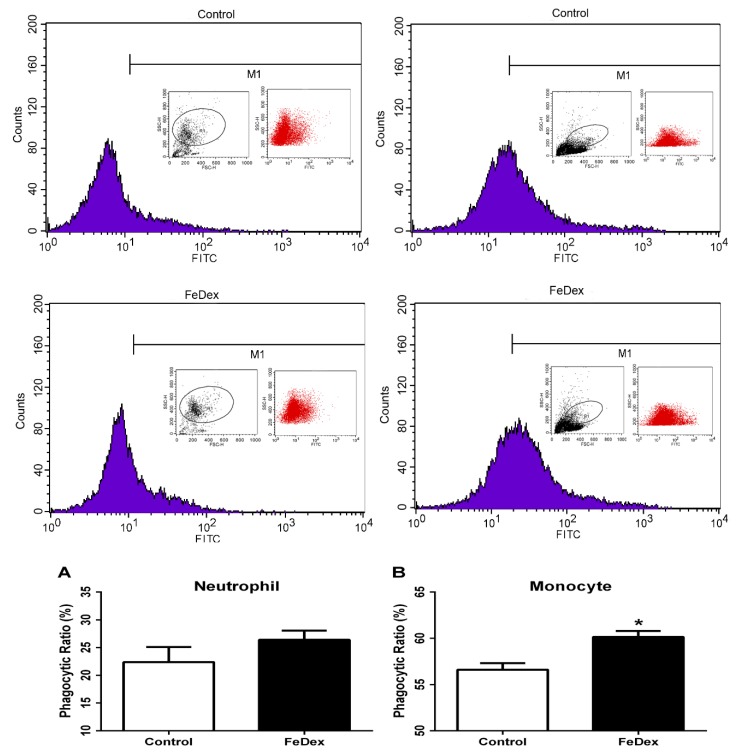
Effect of iron on phagocytic activity of neutrophils (**A**) and monocytes (**B**). Cells were incubated with FITC-dextran at 37 °C for 1 h. The intracellular FITC-dextran was measured by FACS. Data are mean ± SD for four independent experiments (* *p* < 0.05).

Phagocytosis is important for the organism in defense against invading microorganisms and in ridding the body of dead cells [18]. Phagocytes include several cell types such as neutrophils, monocytes, macrophages, dendritic cells, and mast cells. In this study, we determined the phagocytosis of neutrophils and monocytes in PBMC. Iron supplementation caused a significant increase of phagocytosis in monocytes (see Figure 5B). However, in neutrophils, FeDex did not affect the uptake of FITC-labeled dextran (see Figure 5A). The result demonstrated that in vivo iron supplementation increased phagocytosis of monocytes without influencing phagocytosis of neutrophils.

## 4. Discussion

Iron is a crucial element for many central metabolic pathways of the body. It has been confirmed that iron and its homeostasis are closely related to immune development because of its growth-promoting role for immune cells and its interference with cell-mediated immune responses and cytokine activities [19]. Iron deficiency anemia is probably the most prevalent micronutrient deficiency disorder in pigs and the most frequent form of anemia in mammals [2,3]. The main goal of the present study was to investigate the effects of iron deficiency on intestinal immune development in neonatal piglets and to evaluate the benefits of iron supplementation during the first three weeks after birth. 

Along with the nonhuman primate, the neonatal piglet is considered one of the best preclinical models for the human infant [20]. The high degree of similarity in immune variables between humans and pigs (~80%) compared with humans and rodents (~10%) enables studies to focus on immune development or immune responses to infection [21]. Furthermore, neonatal piglets are suitable models for iron deficiency because they are born with limited iron storage and can quickly become iron-deficient without an exogenous iron supplementation [12]. It has been reported that the supplementation of FeDex (60 mg Fe/kg body weight) injected intramuscularly into piglets within several days after birth such as three-day-old piglets will be sufficient to prevent iron deficiency [13]. Once injected into the piglets, iron released from the FeDex is presumed to be slowly removed from the injection site by transferrin and then incorporated into essential body iron or storage since ferritin deposits in organs and tissues [22]. Iron is mostly located as functional iron in hemoglobin [12]. Ferritin serves as the deposit site for cytosolic iron storage and transferrin plays an important role in the uptake of different iron sources [23]. In the present study, the levels of serum ferritin and transferrin in FeDex-supplemented piglets remained stable instead of the decrease of that in control piglets, which suggested that iron supplementation can improve an iron status in piglets. Hepcidin and FPN play a crucial role in regulating systemic iron homeostasis and as a central player in the coordinated response to regulate systemic iron levels [16]. Our results showed that after iron supplementation, the iron level in serum and iron deposits in the liver of neonatal piglets increased rapidly and then decreased gradually with the growth of piglets, which occurred not only as a result of the up-regulated expression of hepcidin followed by lower levels of FPN but also may be due to the need of iron for many life activities.

The early postnatal period is a critical time for gut development. The digestive organs of neonatal piglets grow rapidly throughout the week after birth especially the absorption area of the small intestine. With growth during a suckling period, the duodenum would change in shape and structure such as the increased height of villus, incremental depth of crypt, and enhanced thickness of the intestinal wall [24]. In this study, poorly developed villi of control piglets was possibly due to that iron deficiency blocked the growth and renewal of small intestine cells, which would affect the body’s digestion and absorption capacities [25]. Elevated levels of d-lactic acid and DAO, which are the common signs of intestinal injury, located in the serum of control piglets also indicated the poor barrier function of intestinal mucosa. It was confirmed that iron deficiency could cause modification in intestinal morphology including villous atrophy and alteration in intestinal permeability [2]. However, supplementation with FeDex improved the structures of duodenum and inhibited the exudation of d-lactic acid and DAO, which suggested that iron benefits early intestinal development during the neonatal period. 

Neonates possess a developing immune system. Although the immune system is qualitatively complete at birth, exposures during infancy and early childhood are essential for expansion and priming of adaptive cell populations [26]. Several studies have shown that nutritional imbalance, both deficiency and excess, can have a considerable effect on neonatal immunity at birth and immune maturation in early life [3,26]. Cytokines have effects on and are produced by different T helper (Th) CD4+ cells aht are classified into Th1, Th2, Th3, and Tr1 types [17]. In order to distinguish the cytokines produced by piglets from the maternal origin, we detected the transcripts of pro-inflammatory (IL-6 and IL-1β), Th1 (IFN-γ), and Th3 (TGF-β) cytokines and antimicrobial peptides (pBD-1 and pBD-2) in the ileum of piglets. Our results showed that the immune-associated factors mRNA expressions were low in suckling piglets after birth but rapidly increased starting from day 10. This was in accordance with the principle that active immunity started to play a role in the immune system of suckling piglets. Increased cytokine production by mucosal and systemic immune cells from piglets fed iron suggested a priming of the immune system. Immune cells are better prepared at responding to that challenge by secreting pro-inflammatory cytokines (IL-6, IFN-γ, IL-1β) and antimicrobial peptides (pBD-1, pBD-2) and to recover from the response by secreting anti-inflammatory cyokines (TGF-β). Phagocytosis by macrophages and neutrophils is the organism’s first line of defense against bacterial infection [18]. Our study showed that iron supplementation increased the phagocytosis in monocytes, which suggested that iron was beneficial for immune maturation in piglets. 

## 5. Conclusions

Iron deficiency has adverse effects on intestinal growth and immune development especially in suckling piglets. This study clearly demonstrates that iron supplementation did help improve the iron status, maintain villus structure, keep the transcription of immuno-associated factors, and promote the phagocyte activity of monocytes, which indicated that iron could enhance the intestinal development in neonatal piglets and implied that iron supplemented for newborns is obligatory.
